# Hepatocyte-derived selenoprotein P contributes to pressure overload–induced cardiac remodeling

**DOI:** 10.1016/j.bbrep.2026.102854

**Published:** 2026-10-26

**Authors:** Soichiro Usui, Shinichiro Takashima, Oto Inoue, Chiaki Goten, Yusuke Takeda, Daiki Hashimuko, Kosei Yamaguchi, Yusuke Shinjo, Ayano Nomura, Toshinari Takamura, Shuichi Kaneko, Masayuki Takamura

**Affiliations:** a Department of Cardiovascular Medicine, Kanazawa University Graduate School of Medical Sciences, Kanazawa, Japan; b Department of Endocrinology and Metabolism, Kanazawa University Graduate School of Medical Sciences, Kanazawa, Japan; c Information-Based Medicine Development, Kanazawa University Graduate School of Medical Sciences, Kanazawa, Japan

**Keywords:** Selenoprotein P, Hepatokine, Liver-heart axis, Pressure overload, Cardiac remodeling, Heart failure

## Abstract

**Background:**

Heart failure (HF) is increasingly recognized as a systemic disorder involving inter-organ communication. However, the role of the liver-heart axis in cardiac remodeling remains poorly understood. Selenoprotein P (Selenop), a hepatokine involved in systemic metabolic regulation, has been associated with HF, but its causal and tissue-specific contributions to cardiac remodeling remain unclear.

**Methods and results:**

We investigated the role of Selenop in pressure overload–induced cardiac remodeling using a transverse aortic constriction (TAC) in global and hepatocyte-specific Selenop knockout mice. TAC increased hepatic Selenop expression and circulating Selenop protein without altering cardiac Selenop expression. These changes were accompanied by reduced hepatic Akt and FoxO1 phosphorylation. Global Selenop deficiency improved survival and attenuated TAC-induced cardiac hypertrophy, pulmonary congestion, systolic dysfunction, and interstitial fibrosis. Transcriptomic and quantitative RT-PCR analyses showed that Selenop deficiency reduced cardiac Tgfb1 expression and attenuated TGF-β-associated profibrotic genes, including extracellular matrix-related genes. Hepatocyte-specific Selenop deletion markedly reduced circulating Selenop and attenuated TAC-induced cardiac hypertrophy, pulmonary congestion, systolic dysfunction, and profibrotic gene expression.

**Conclusions:**

Hepatocyte-derived Selenop contributes to adverse cardiac remodeling under pressure overload and is associated with a TGF-β-related profibrotic transcriptional response in the heart. These findings support Selenop-mediated liver-heart communication as a potential mechanism of HF progression.

## Highlights

• Pressure overload increases hepatic expression and circulating selenoprotein P.
• Global selenoprotein P deficiency attenuates adverse cardiac remodeling.
• Hepatocyte-specific deletion reduces circulating selenoprotein P.
• Hepatocyte-derived selenoprotein P contributes to profibrotic cardiac responses.

## 1 Introduction

Heart failure (HF) remains a major global health burden with high morbidity and mortality rates [1]. Although HF arises from diverse etiologies, including ischemic injury, valvular dysfunction, and cardiomyopathies, the molecular mechanisms underlying ventricular remodeling are multifactorial and involve oxidative stress, inflammation, mitochondrial dysfunction, and neurohormonal activation. Increasingly, HF is recognized as a systemic syndrome in which inter-organ communication plays an important role in disease progression. Consistent with this concept, non-cardiovascular comorbidities frequently exacerbate HF and are independently associated with poor outcomes.

Among extracardiac organ systems implicated in HF pathophysiology, the heart–kidney and heart–brain axes have been well characterized [2]. More recently, the liver-heart axis has emerged as another potentially important contributor, as liver dysfunction is common and prognostically significant in HF. Under physiological conditions, the liver receives a substantial fraction of cardiac output. In HF, reduced cardiac output can cause hepatic hypoperfusion, whereas elevated central venous pressure can lead to hepatic congestion. These hemodynamic disturbances contribute to liver injury, which is reflected by abnormalities in hepatobiliary biomarkers and is associated with HF severity and adverse outcomes [3]. In addition, liver stiffness assessed by elastography correlates with right-sided filling pressure and predicts prognosis in patients with HF [4]. Experimental studies have further demonstrated that cardiac stress, including myocardial ischemia [5] and rapid atrial pacing [6], induces changes in hepatic gene expression, supporting the concept that the liver actively responds to cardiac injury and dysfunction.

Beyond its metabolic function, the liver acts as an endocrine organ by secreting hepatokines that regulate systemic metabolism and distant-organ function [7]. Selenoprotein P (Selenop), the major selenium-transport protein in the circulation, is produced predominantly by hepatocytes. Selenop has been implicated in insulin resistance and metabolic disorders, including type 2 diabetes [8]. In experimental models, endogenous Selenop impairs angiogenesis, and global Selenop deletion reduces myocardial injury following ischemia–reperfusion [9], suggesting that its biological effects extend beyond the regulation of systemic metabolism.

Clinically, elevated circulating Selenop levels have been associated with adverse outcomes in patients with HF, including cardiac death and HF hospitalization [10]. In contrast, lower baseline Selenop levels in population-based cohorts have been linked to a higher risk of incident HF [11]. These apparently divergent findings suggest that the relationship between Selenop and HF may be context dependent and remains incompletely understood. In particular, it is unclear whether elevated circulating Selenop actively contributes to HF progression or represents a biomarker of systemic metabolic and hemodynamic stress.

Despite these clinical and experimental observations, the causal role and tissue-specific contribution of Selenop in HF remain poorly defined. In particular, whether Selenop contributes to pressure overload-induced cardiac remodeling and whether hepatocyte-derived Selenop mediates this response have not been established. Given the growing recognition of the liver-heart axis in HF pathophysiology, we hypothesized that hepatocyte-derived Selenop contributes to maladaptive cardiac remodeling under hemodynamic stress.

In the present study, we investigated the role of Selenop in pressure overload-induced cardiac remodeling using both global and hepatocyte-specific Selenop knockout mouse models. We examined the effects of Selenop deficiency on cardiac hypertrophy, fibrosis, and systolic dysfunction and assessed changes in hepatic and circulating Selenop following pressure overload. We further explored the hepatic signaling pathways associated with Selenop induction and the cardiac profibrotic pathways associated with its effects. Our findings provide evidence that hepatocyte-derived Selenop contributes to adverse cardiac remodeling under pressure overload and identify Selenop-mediated liver-heart communication as a potential pathway involved in HF progression.

## 2 Methods

### 2.1 Animals

All animal experiments were conducted in accordance with the Guidelines for the Care and Use of Laboratory Animals of Kanazawa University and were approved by the Animal Ethics Committee of Kanazawa University (Approval No. AP24-0262). Global Selenop knockout (KO) mice were generated as previously described [12], and wild-type littermates (WT) were used as controls. Floxed Selenop mice were generated as previously described [13]. Liver-specific Selenop knockout (L-Selenop KO) mice were generated by crossing Selenop^fl/fl^ mice with albumin promoter-driven Cre (Alb-Cre) transgenic mice. The Alb-Cre mice were kindly provided by Dr. Hiroshi Inoue (Kanazawa University, Japan). L-Selenop KO mice were maintained on a mixed C57BL/6 and 129 genetic background after more than five generations of backcrossing to C57BL/6 mice. Selenop^fl/fl^ littermates lacking the Alb-Cre transgene (Control) were phenotypically indistinguishable and used as controls. Genotyping was performed by PCR. Only male mice were used in this study. Mice were maintained on a standard rodent diet (CRF-1; Oriental Yeast, Tokyo, Japan) containing 0.45 mg/kg of selenium and free access to food and water. At the end of each experiment, animals were euthanized by cervical dislocation after isoflurane inhalation (2-2.5 vol%).

### 2.2 Transverse aortic constriction

Pressure overload (PO) was induced by transverse aortic constriction (TAC) as previously described [14], [15]. Briefly, mice were anesthetized by intraperitoneal injection of a mixture of three anesthetic agents (0.3 mg/kg medetomidine, 4 mg/kg midazolam, and 5 mg/kg butorphanol) and mechanically ventilated. The chest was opened at the second intercostal space, and the transverse thoracic aorta was constricted against a 28-gauge needle using a 7-0 braided polyester suture. The needle was then removed. Sham-operated mice underwent the same procedure without aortic ligation. The degree of aortic constriction was assessed by measurement of aortic flow velocity after TAC.

### 2.3 Survival analysis

Survival was monitored for 14 days after TAC in Selenop-deficient and wild-type (WT) mice. Mice were inspected daily, and deceased mice were recorded. Survival curves were generated using the Kaplan-Meier method and compared using the log-rank test.

### 2.4 Echocardiography

Cardiac function was assessed by transthoracic echocardiography 14 days after TAC. Mice were lightly anesthetized with 1.5% isoflurane, maintaining heart rates between 400 and 500 bpm. Two-dimensional short-axis views were obtained using a high-resolution ultrasound system (Vevo 2100; VisualSonics, Toronto, Canada) equipped with a 40-MHz transducer. Left ventricular dimensions and systolic function, including left ventricular ejection fraction (LVEF), were determined using Vevo analysis software. All measurements were performed by an investigator blinded to experimental groups.

### 2.5 Histological analysis

Histological procedures were performed as described previously [16]. Hearts were fixed in 10% neutral-buffered formalin, embedded in paraffin, and sectioned at 6 μm thickness. Cardiomyocyte cross-sectional area was assessed using hematoxylin and eosin (H&E)-stained sections. Cardiomyocytes with clearly defined cell borders and approximately circular profiles were selected, and 100 cardiomyocytes per section were analyzed using ImageJ software (National Institutes of Health, Bethesda, MD, USA). Myocardial fibrosis was assessed using Picrosirius Red staining. Representative whole heart, interstitial, and perivascular regions were examined. Quantitative analysis was performed for interstitial fibrosis only. Digital images were analyzed using ImageJ software. Collagen-positive areas within the myocardial interstitium were measured and expressed as a percentage of the analyzed myocardial tissue area.

### 2.6 Quantitative real-time PCR (qRT–PCR)

qRT–PCR was conducted as previously reported [17]. Total RNA was extracted from cardiac tissue using an RNeasy Fibrous Tissue Mini Kit (Qiagen). cDNA was synthesized and analyzed using TaqMan gene expression assays and a Universal PCR Master Mix (Applied Biosystems) on an ABI Prism 7900HT Sequence Detection System. The following TaqMan probes were used: Genes of interest: Nppb (Mm01255770_g1), Myh7 (Mm01319006_g1), Myh6 (Mm01313844_mH), Col1a1 (Mm00801666_g1), Col3a1 (Mm01254476_m1), Tgfb1 (Mm00441726_m1), Selenop (Mm00486048_m1), Foxo1 (Mm00490671_m1), Foxo3 (Mm01185722_m1), Postn (Mm01284919_m1), Ctgf (Mm01192933_g1), Fn1(Mm01256744_m1), Housekeeping gene: Gapdh (Mm99999915_g1)

### 2.7 Western blot analysis

Western blot analysis was performed as previously described [17]. Briefly, liver tissues were homogenized in lysis buffer supplemented with protease and phosphatase inhibitors, and equal amounts of protein were subjected to SDS-PAGE and immunoblotting. Primary antibodies against phosphorylated FoxO1 (p-FoxO1; Ser256), total FoxO1, phosphorylated Akt (p-Akt; Ser473), and total Akt were obtained from Cell Signaling Technology, and the antibody against GAPDH was obtained from Thermo Fisher Scientific. Protein bands were visualized using enhanced chemiluminescence (ECL), and images were acquired and analyzed using a ChemiDoc imaging system (BioRad Laboratories).

### 2.8 Serum Selenop immunoprecipitation and immunoblotting

Circulating Selenop protein was assessed by immunoprecipitation followed by immunoblotting. One microliter of serum from each mouse was diluted in 500 μL of immunoprecipitation buffer consisting of PBS(−) containing 0.1% NP-40. Selenop was immunoprecipitated using Selenoprotein P (B-9) agarose-conjugated antibody (Santa Cruz Biotechnology). After washing with immunoprecipitation buffer, the immunoprecipitated proteins were eluted in 2× SDS sample buffer and subjected to SDS-PAGE and immunoblotting as described above using an anti-Selenop antibody (Thermo Fisher Scientific). Serum from global Selenop KO mice was used as a negative control to confirm the specificity of the detected Selenop band. Band intensity was quantified using the ChemiDoc imaging system and expressed relative to the corresponding control group.

### 2.9 Microarray analysis

Total RNA from mouse hearts was isolated using an RNeasy Fibrous Tissue Mini Kit (Qiagen). 100 ng of RNA was amplified, labeled, and hybridized to Affymetrix Mouse Gene 1.0 ST arrays (Affymetrix) following the manufacturer's instructions [17]. Data were extracted and normalized using the Affymetrix Expression Console and processed with BRB-ArrayTools. Probes with absent detection calls were excluded. Remaining data were log-transformed and median-normalized. Differential gene expression between TAC-operated WT and Selenop KO hearts was assessed by class comparison. Genes showing an absolute fold change of >1.5 between the two groups were selected for hierarchical clustering. Hierarchical clustering was performed using average linkage with centered correlation. Functional enrichment analysis was performed using MetaCore (Clarivate Analytics) to identify biological pathways differentially represented between the experimental groups. The microarray data generated in this study have been deposited in the NCBI Gene Expression Omnibus (GEO) under accession number GSE297651.

### 2.10 Statistical analysis

Data are presented as mean ± standard error of the mean (SEM). Comparisons between two groups were performed using an unpaired two-tailed Student's t-test. For experiments involving genotype and surgical treatment as two independent factors, two-way analysis of variance (ANOVA) followed by Bonferroni's multiple-comparison test was used. Survival curves were analyzed using the Kaplan-Meier method and compared using the log-rank test. A two-sided P value < 0.05 was considered statistically significant. Statistical analyses were performed using GraphPad Prism (GraphPad Software).

## 3 Results

### 3.1 Selenop deficiency attenuates pressure overload-induced cardiac hypertrophy

To investigate the role of Selenop in pressure overload (PO)-induced cardiac remodeling, we subjected global Selenop knockout (KO) and wild-type (WT) mice to transverse aortic constriction (TAC). Aortic flow velocity after TAC did not differ significantly between WT and Selenop KO mice, indicating a comparable degree of aortic constriction between the two groups (Fig. 1A). During the 14-day observation period, mortality after TAC was significantly lower in Selenop KO than in WT mice (Fig. 1B). Fourteen days after TAC, the increase in the left ventricular weight to tibial length (LVW/TL) ratio was significantly attenuated in Selenop KO mice compared with WT mice (Fig. 1C). Similarly, TAC-induced cardiomyocyte hypertrophy, assessed by LV cardiomyocyte cross-sectional area, was significantly reduced in Selenop KO mice (Fig. 1D). TAC also markedly increased the expression of the fetal gene Nppb and the Myh7/Myh6 mRNA ratio in WT hearts, whereas these responses were significantly attenuated in Selenop KO hearts (Fig. 1E and F). These findings indicate that Selenop deficiency attenuates cardiac hypertrophy and fetal gene reprogramming in response to PO.

**Fig. 1:**
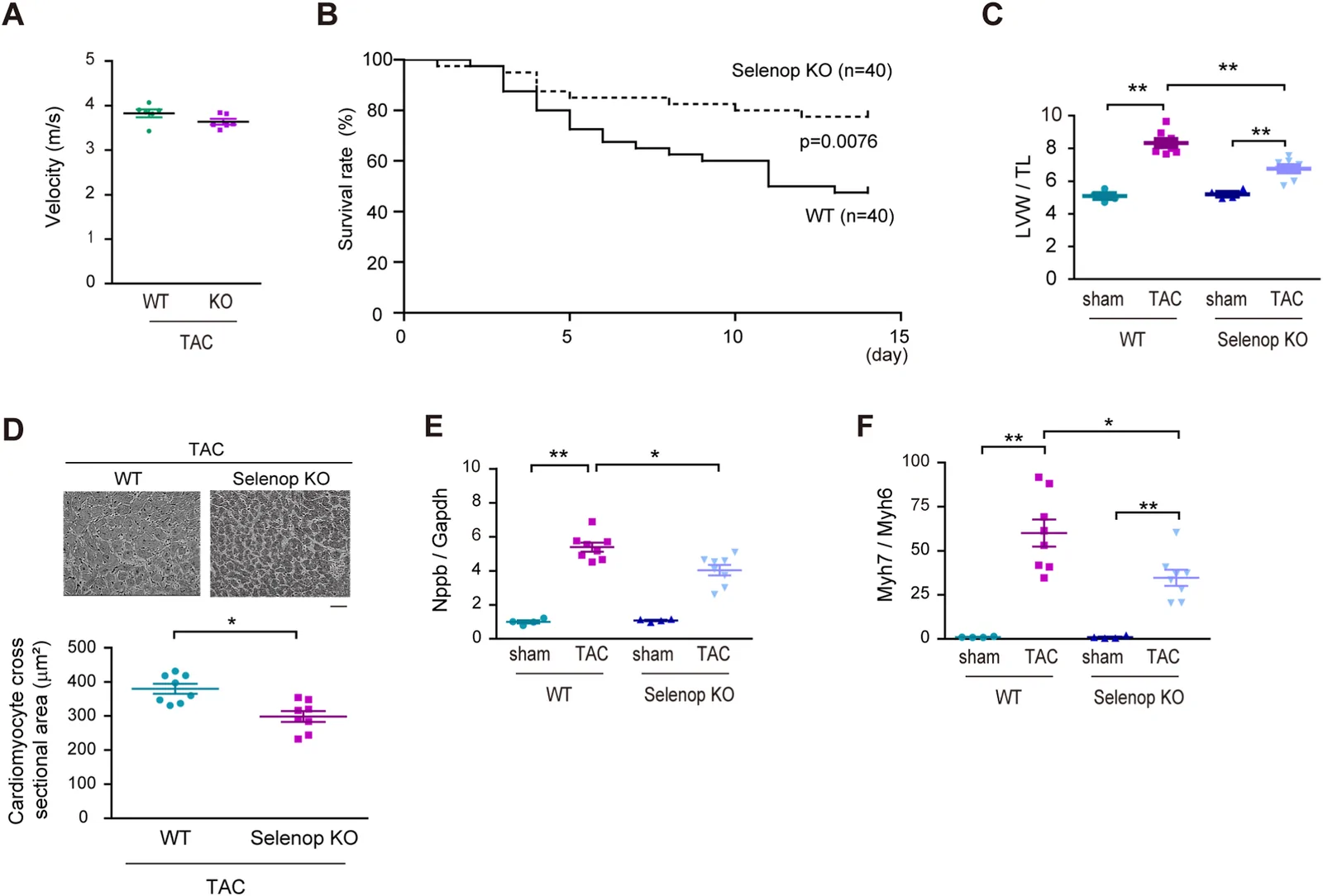
Selenoprotein P (Selenop) deficiency attenuates pressure overload (PO)-induced cardiac hypertrophy. A, Aortic flow velocity in wild-type (WT) and Selenop knockout (KO) mice after transverse aortic constriction (TAC). (n = 6 per group). B, Kaplan–Meier survival curves of WT (n = 40) and Selenop KO (n = 40) mice after TAC. C, Left ventricular weight to tibial length (LVW/TL) ratio in sham- and TAC-operated mice (n = 4 [sham], n = 8 [TAC]). D, Representative hematoxylin and eosin-stained myocardial sections (upper) and quantitative analysis (lower) (n = 8 [TAC]). Scale bar: 200 μm. E, F, Cardiac mRNA expression of Nppb (E) and the Myh7/Myh6 ratio (F) (n = 4 [sham], n = 8 [TAC]). *P < 0.05, **P < 0.01.

### 3.2 Selenop deficiency preserves cardiac function and attenuates myocardial fibrosis after pressure overload

We next examined the effects of Selenop deficiency on cardiac dysfunction and fibrosis after TAC. Fourteen days after TAC, the lung weight to TL ratio (Lung W/TL), an index of pulmonary congestion, was significantly lower in Selenop KO mice than in WT mice (Fig. 2A). Echocardiographic assessment showed that TAC-induced reduction in left ventricular ejection fraction (LVEF) was significantly attenuated in Selenop KO mice (Fig. 2B). Histological analysis using Picrosirius Red staining demonstrated less collagen deposition in Selenop KO hearts after TAC, and quantitative analysis confirmed a significant reduction in collagen-positive interstitial myocardial area compared with WT hearts (Fig. 2C). Consistent with these histological findings, TAC-induced increases in the cardiac expression of the extracellular matrix genes Col1a1 and Col3a1 were significantly attenuated in Selenop KO mice (Fig. 2D and E). Together, these findings indicate that Selenop deficiency preserves systolic function, reduces pulmonary congestion, and attenuates myocardial fibrotic remodeling following PO.

**Fig. 2:**
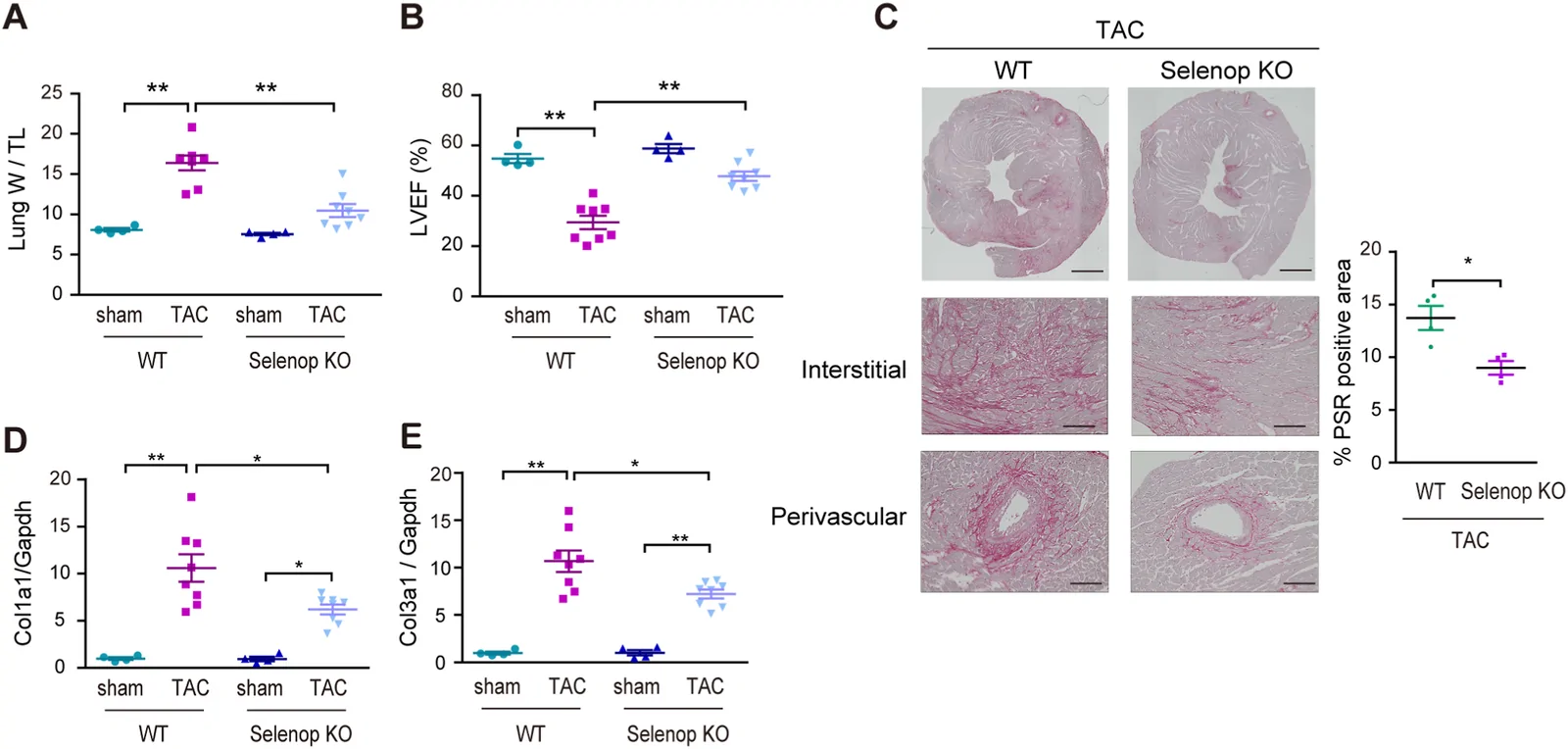
Selenop deficiency preserves cardiac function and attenuates myocardial fibrosis following TAC. A, Lung weight to TL (Lung W/TL) ratio in sham- and TAC-operated WT and Selenop KO mice (n = 4 [sham], n = 8 [TAC]). B, Left ventricular ejection fraction (LVEF) assessed by echocardiography (n = 4 [sham], n = 8 [TAC]). C, Representative images of Picrosirius Red (PSR)-stained whole-heart (upper), interstitial (middle), and perivascular (lower) regions from WT and Selenop KO mice 14 days post-TAC (left), and quantification of interstitial fibrosis (right). Scale bars, 1000 μm (upper), 200 μm (middle and lower). D, E, Cardiac mRNA expression of Col1a1 (D), and Col3a1 (E) (n = 4 [sham], n = 8 [TAC]). *P < 0.05, **P < 0.01.

### 3.3 Selenop deficiency attenuates TGF-β-associated profibrotic gene expression after TAC

To further investigate the molecular pathway associated with the protective effects of Selenop deficiency, we performed microarray analysis of heart tissue from TAC-operated WT and Selenop KO mice. Hierarchical clustering of 645 filtered genes showing an absolute fold change of > 1.5 between TAC-operated WT and Selenop KO hearts revealed distinct gene expression profiles between WT and Selenop KO hearts after TAC (Fig. 3A). Class comparison analysis (P < 0.0001) identified enrichment of pathways associated with fibroblast/myofibroblast proliferation and TGF-β related signaling in WT compared with Selenop KO hearts (Fig. 3B). Consistent with the transcriptomic analysis, cardiac Tgfb1 expression was significantly lower in Selenop KO mice after TAC than in WT mice (Fig. 3C). In addition, TAC-induced expression of the profibrotic genes Postn, Ctgf, and Fn1 was significantly attenuated in Selenop KO hearts (Fig. 3D-F). Together with the reduced expression of Col1a1 and Col3a1, these findings demonstrated that Selenop deficiency attenuates TGF-β-associated profibrotic gene expression in the PO heart.

**Fig. 3:**
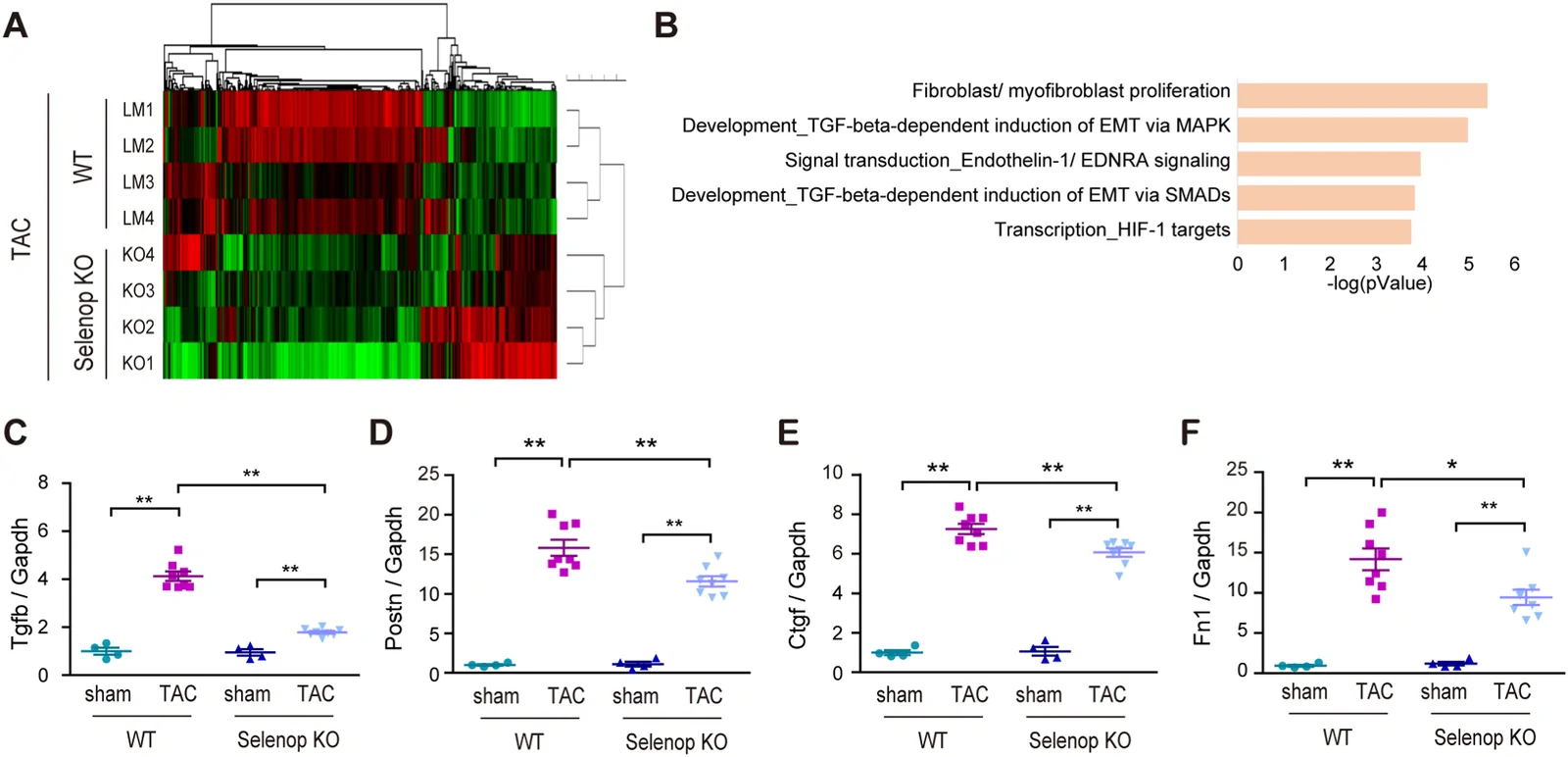
Selenop deficiency attenuates TGF-β-associated profibrotic gene expression following TAC. A, Hierarchical clustering of cardiac gene expression in TAC-operated WT and Selenop KO mice (n = 4). B, Pathway analysis of differentially expressed genes between WT and Selenop KO hearts after TAC. C–F, Cardiac mRNA expression of Tgfb1 (C), Postn (D), Ctgf (E), and Fn1 (F) in sham- and TAC-operated WT and Selenop KO mice (n = 4 [sham], n = 8 [TAC]). *P < 0.05, **P < 0.01.

### 3.4 PO increases hepatic and circulating selenop and is associated with reduced hepatic Akt and FoxO1 phosphorylation

To determine the source and regulation of Selenop after PO, we examined Selenop expression in heart and liver 14 days after TAC. Cardiac Selenop mRNA expression was unchanged after TAC (Fig. 4A), whereas hepatic Selenop expression was significantly increased (Fig. 4B). Consistent with the increase in hepatic expression, immunoprecipitation followed by immunoblotting demonstrated increased circulating Selenop protein in WT mice after TAC (Fig. 4C). The specificity of the detected serum Selenop signal was supported by its absence in serum from global Selenop KO mice.

**Fig. 4:**
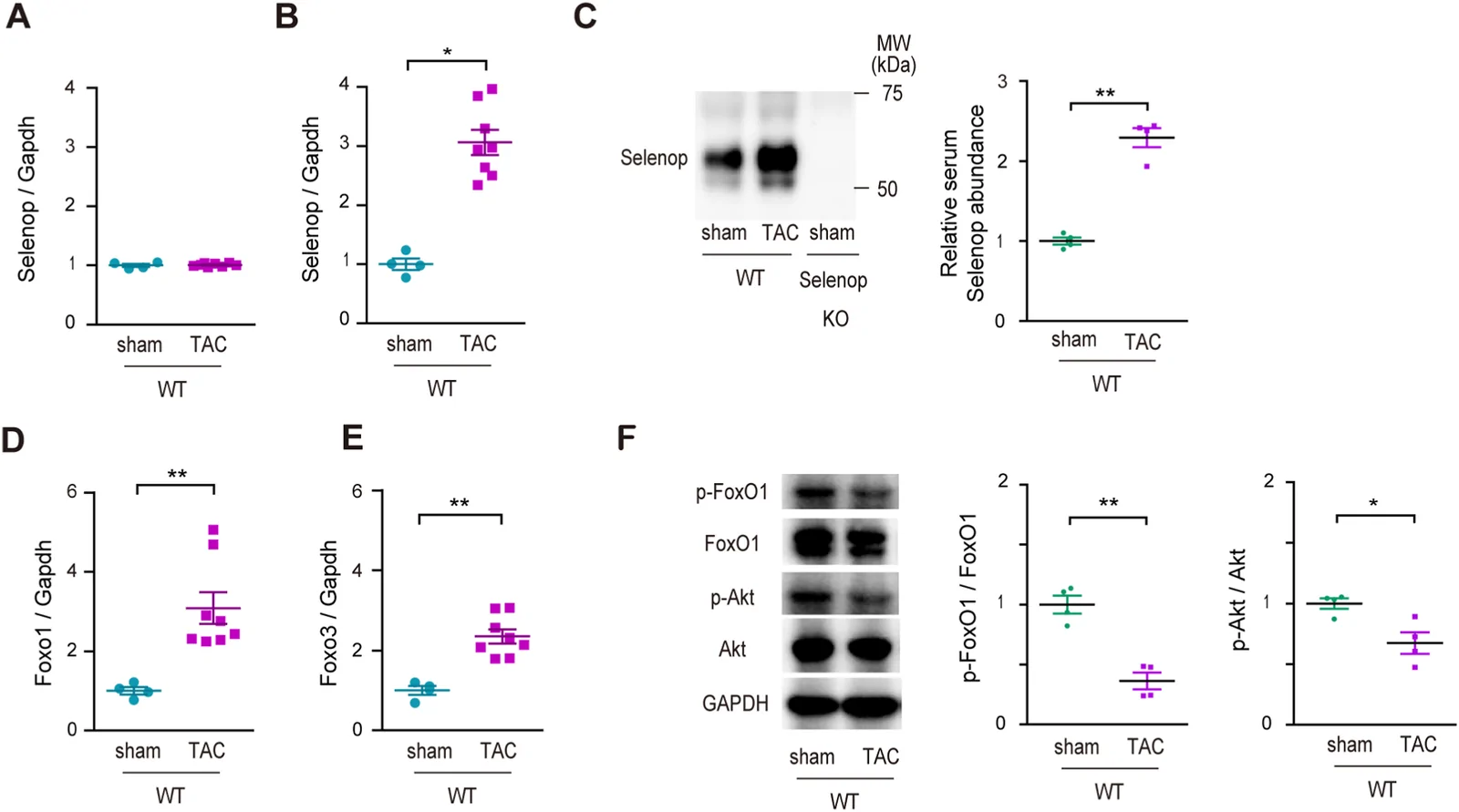
TAC increases hepatic and circulating Selenop and is associated with reduced hepatic Akt and FoxO1 phosphorylation. A, B, Selenop mRNA expression in the heart (A) and liver (B) of WT mice 14 days after sham or TAC surgery (n = 4 [sham], n = 8 [TAC]). C, Representative immunoblot and quantitative analysis of serum Selenop in WT mice 14 days after sham or TAC surgery (n = 4 per group). Serum from global Selenop KO mice was included as a negative control. D, E, Hepatic mRNA expression of Foxo1 (D) and Foxo3 (E) 14 days after sham or TAC surgery (n = 4 [sham], n = 8 [TAC]). F, Representative immunoblots of phosphorylated FoxO1(p-FoxO1; Ser256), total FoxO1, phosphorylated Akt (p-Akt; Ser473), total Akt, and GAPDH in liver 14 days after sham or TAC surgery (left), and quantitative analysis of the p-FoxO1/FoxO1 and p-Akt/Akt ratios (right) (n = 4 per group). *P < 0.05, **P < 0.01.

Because Foxo transcription factors are involved in the transcriptional regulation of Selenop, we next examined hepatic Foxo1 and Foxo3 expression. Both Foxo1 and Foxo3 mRNA levels were significantly increased in the liver after TAC (Fig. 4D and E). In parallel, hepatic phosphorylation of FoxO1 and Akt was reduced after TAC, as reflected by decreases in the p-FoxO1/FoxO1 and p-Akt/Akt ratios (Fig. 4F). These findings show that PO increases hepatic and circulating Selenop and is accompanied by reduced hepatic Akt and FoxO1 phosphorylation.

### 3.5 Hepatocyte-specific selenop deletion reduces circulating Selenop and attenuates PO-induced cardiac remodeling

To directly assess the contribution of hepatocyte-derived Selenop to cardiac remodeling, we generated liver-specific Selenop KO (L-Selenop KO) mice by crossing Selenop^fl/fl^ mice with Alb-Cre transgenic mice (Fig. 5A). Selenop mRNA expression was markedly reduced in liver of L-Selenop KO mice, whereas cardiac Selenop expression was unchanged (Fig. 5B and C). Consistent with the liver being a major source of circulating Selenop, serum Selenop protein abundance was also markedly reduced in L-Selenop KO mice (Fig. 5D).

**Fig. 5:**
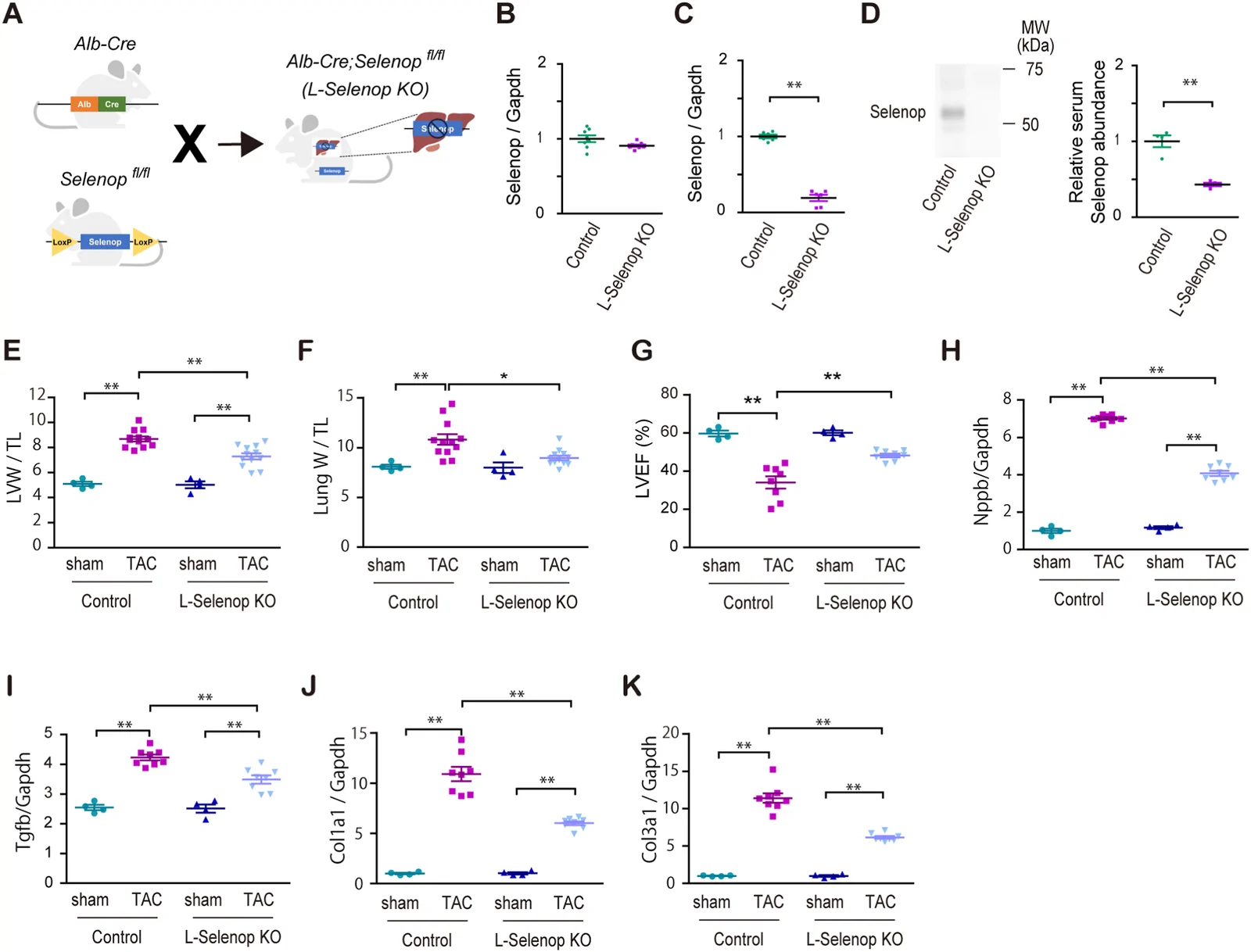
Hepatocyte-specific Selenop deletion reduces circulating Selenop and attenuates cardiac remodeling following TAC. A, Schematic representation of the generation of liver-specific Selenop (L-Selenop) KO mice (Alb-Cre; Selenop^fl/fl^). The schematic was created with BioRender.com. B, C, Selenop mRNA expression in the heart (B) and liver (C) of Control and L-Selenop KO mice under baseline conditions. (n = 8 per group). D, Representative immunoblot and quantitative analysis of serum Selenop in Control and L-Selenop KO mice under baseline conditions (n = 4 per group). E, F, LVW/TL (E) and lung W/TL (F) ratios in sham- and TAC-operated Control and L-Selenop KO mice (n = 4 [sham], n = 12 [TAC]). G, LVEF assessed by echocardiography (n = 4 [sham], n = 8 [TAC]). H–K, Cardiac mRNA expression of Nppb (H), Tgfb1 (I), Col1a1 (J), and Col3a1 (K) in sham- and TAC-operated Control and L-Selenop KO mice (n = 4 [sham], n = 8 [TAC]). *P < 0.05, **P < 0.01.

Following TAC, the increase in LVW/TL was significantly attenuated in L-Selenop KO mice compared with control mice (Fig. 5E). Lung W/TL was also significantly lower in L-Selenop KO mice after TAC (Fig. 5F). Furthermore, the TAC-induced reduction in LVEF was significantly attenuated in L-Selenop KO mice (Fig. 5G). At the molecular level, TAC-induced increases in Nppb, Tgfb1, Col1a1, and Col3a1 expression were significantly attenuated in L-Selenop KO hearts (Fig. 5H-K). These findings demonstrated that hepatocyte-derived Selenop contributes to PO-induced cardiac hypertrophy, pulmonary congestion, systolic dysfunction, and profibrotic gene expression.

## 4 Discussion

The present study demonstrates that selenoprotein P (Selenop) contributes to pressure overload (PO)-induced cardiac remodeling. We show that PO selectively upregulates Selenop expression in the liver, with no corresponding increase in the heart, and increases circulating Selenop protein. Global Selenop deficiency attenuates cardiac hypertrophy, myocardial fibrosis, pulmonary congestion, and systolic dysfunction after transverse aortic constriction (TAC). Moreover, hepatocyte-specific deletion of Selenop markedly reduced circulating Selenop and significantly attenuated PO-induced cardiac hypertrophy, pulmonary congestion, systolic dysfunction, and profibrotic gene expression. These findings identify hepatocyte-derived Selenop as a contributor to adverse cardiac remodeling and highlight a previously unrecognized liver-heart axis in the pathogenesis of heart failure (HF).

HF is increasingly recognized as a systemic syndrome involving inter-organ communication [18]. Among non-cardiac organs, the liver plays a central role in metabolic regulation and is highly sensitive to hemodynamic stress, including hypoperfusion and congestion [19]. Liver dysfunction is common in HF and is associated with disease severity and adverse outcomes. In this context, hepatokines have emerged as important mediators linking hepatic stress responses to cardiovascular pathology [20], [21]. Our findings extend this concept by identifying Selenop as a liver-derived factor that contributes to PO-induced cardiac remodeling. The increases in circulating Selenop after TAC, together with its marked reduction following hepatocyte-specific Selenop deletion, further support the liver as a major source of circulating Selenop under PO. These findings suggest that hepatic responses to cardiac hemodynamic stress may not merely reflect secondary organ dysfunction but may actively modulate cardiac remodeling through hepatokine-mediated inter-organ communication. Importantly, the protective effects of hepatocyte-specific Selenop deletion indicate that, in this model, increased circulating Selenop is not solely a biomarker of hepatic or hemodynamic stress but contributes to adverse cardiac remodeling. Nevertheless, circulating Selenop may also reflect hepatic hypoperfusion or systemic stress in patients with established HF [10]. The contrast between our findings and population-based observations associating lower baseline Selenop levels with incident HF [11] may reflect differences in disease stage, selenium status, and the biological context in which circulating Selenop is measured. Further studies are required to determine whether these context-dependent associations also reflect differences in the biological actions of Selenop.

Insulin resistance (IR), even in the absence of overt diabetes, is a recognized risk factor for HF [22]. Proposed mechanisms linking IR to HF include hyperinsulinemia, fluid retention, sympathetic activation, and enhanced angiotensin II signaling [23]. Selenop, a hepatokine involved in systemic metabolism, has been shown to induce IR and promote metabolic dysfunction [24]. In addition, excess Selenop impairs angiogenesis and exacerbates myocardial injury in ischemic models [9]. Our findings extend these observations to PO-induced HF by demonstrating that Selenop deficiency attenuates cardiac remodeling under hemodynamic stress. Notably, TAC increased hepatic Foxo1 and Foxo3 expression and reduced hepatic Akt and FoxO1 phosphorylation. Because Akt-mediated phosphorylation suppresses Foxo transcriptional activity, these findings are consistent with the possibility that reduced Akt-FoxO1 signaling contributes to increased hepatic Selenop expression after TAC. Although hepatic perfusion was not directly assessed, hemodynamic alteration induced by TAC may have contributed to this hepatic response. Consistent with this possibility, elevated circulating Selenop levels have been associated with hepatic hypoperfusion in patients with HF [10]. We therefore speculate that hepatic hypoperfusion may contribute to reduced Akt-mediated FoxO1 phosphorylation and increased Selenop expression. However, the present study did not assess FoxO1 nuclear localization or its direct binding to the Selenop promoter, and therefore does not establish a causal role for this pathway. Importantly, despite the absence of a TAC-induced increase in cardiac Selenop expression, hepatocyte-specific deletion of Selenop was sufficient to confer cardioprotection, underscoring the systemic impact of hepatocyte-derived Selenop.

Cardiac fibrosis is a key determinant of HF progression and is characterized by excessive extracellular matrix (ECM) deposition [25]. Persistent activation of cardiac fibroblasts promotes ECM accumulation, impairs myocardial compliance, and contributes to systolic dysfunction [26]. In the present study, global Selenop deficiency significantly attenuated PO-induced interstitial fibrosis. Transcriptomic and qRT-PCR analyses consistently demonstrated downregulation of profibrotic pathways and genes, particularly those associated with TGF-β signaling, including Tgfb1, Col1a1, Col3a1, Postn, Ctgf, and Fn1. These findings suggest that hepatocyte-derived Selenop promotes cardiac fibrosis through mechanisms associated with a TGF-β-related profibrotic transcriptional program, providing a potential mechanistic link between hepatic stress responses and myocardial remodeling. However, because canonical downstream mediators such as SMAD2/3 phosphorylation were not examined, whether Selenop directly activates TGF-β signaling in cardiac fibroblasts remains to be determined.

This study has several limitations. First, although reduced hepatic Akt and FoxO1 phosphorylation was associated with increased hepatic Selenop expression after TAC, the causal role of Akt-FoxO1 pathway was not directly examined. Second, although we identified TGF-β-associated pathways as potential mediators, the precise molecular mechanisms linking circulating Selenop to cardiac fibroblast activation remain to be elucidated. Third, our findings are based primarily on murine models of pressure overload and may not fully capture the heterogeneity of human HF. Fourth, the magnitude of attenuation of TAC-induced phenotypes differed modestly between the global and hepatocyte-specific Selenop KO cohorts. Because these models were studied in independent surgical series and differed in genetic background (C57BL/6 for global Selenop KO mice versus mixed C57BL/6 and 129 for L-Selenop KO mice), their effect sizes cannot be directly compared. These experimental differences may have contributed to the observed variation in phenotype magnitude. Finally, we did not assess the reversibility of cardiac remodeling following Selenop inhibition, which will be important for therapeutic translation.

In conclusion, PO increases hepatic Selenop expression and circulating Selenop abundance in association with reduced hepatic Akt and FoxO1 phosphorylation. Global and hepatocyte-specific deletion of Selenop attenuated PO-induced cardiac remodeling and suppressed TGF-β-associated profibrotic gene expression. These findings identify hepatocyte-derived Selenop as a component of maladaptive liver-heart communication under hemodynamic stress and suggest that modulation of the Selenop pathway may represent a potential strategy for preventing HF progression.

## CRediT authorship contribution statement

**Soichiro Usui:** Writing – review & editing, Writing – original draft, Project administration, Investigation, Conceptualization. **Shinichiro Takashima:** Writing – review & editing, Validation. **Oto Inoue:** Formal analysis, Data curation. **Chiaki Goten:** Formal analysis, Data curation. **Yusuke Takeda:** Validation, Investigation. **Daiki Hashimuko:** Validation, Investigation. **Kosei Yamaguchi:** Visualization. **Yusuke Shinjo:** Visualization. **Ayano Nomura:** Visualization, Validation, Investigation. **Toshinari Takamura:** Writing – review & editing, Supervision, Resources, Conceptualization. **Shuichi Kaneko:** Supervision, Funding acquisition, Conceptualization. **Masayuki Takamura:** Writing – review & editing, Supervision, Project administration, Funding acquisition, Conceptualization.

## Ethics declaration

This study was conducted in accordance with the ARRIVE (Animal Research: Reporting of In Vivo Experiments) guidelines. This study was approved by the Animal Ethics Committee of Kanazawa University. (Approval No. AP24-0262)

## Declaration of generative AI and AI-assisted technologies in the writing process

During the preparation of this work, the authors used ChatGPT (OpenAI) to improve the language and readability of the manuscript. The authors reviewed and edited the output as needed and take full responsibility for the content of the published article.

## Sources of funding

This work was supported by the following grants: 10.13039/501100001691JSPS KAKENHI, Japan 15K09135 (S.U.).

## Declaration of competing interest

The authors declare that they have no competing interests.

## Data Availability

The microarray data generated in this study have been deposited in the NCBI Gene Expression Omnibus (GEO) under accession number GSE297651. All other data supporting the findings of this study are available from the corresponding author upon reasonable request.
